# Genome sequence and annotation of *Arthrobacter globiformis* phage Vulpecula (AS1) isolated from soil in Dahlonega, Georgia

**DOI:** 10.1128/mra.00090-24

**Published:** 2024-02-22

**Authors:** Lily Chuhran, Chase Whitlow, Carson Teems, Alison Kanak, Shane A. Webb

**Affiliations:** 1 Department of Biology, University of North Georgia, Dahlonega, Georgia, USA; Queens College Department of Biology, Queens, New York, USA

**Keywords:** viral bioinformatics, genome annotation

## Abstract

Vulpecula, a temperate bacteriophage collected from soil in Dahlonega, Georgia using host *Arthrobacter globiformis*, is an AS1 subcluster virus of 37,766 bp (67.7% GC). Genome annotation suggests 64 open reading frames, no predicted tRNA genes, and ~98% sequence similarity to AS1 phages Ruchi (from GA) and Jamun (New Hampshire).

## ANNOUNCEMENT

Bacteriophages represent a potential for weaponization in the fight against multidrug resistance (bacteriophage therapy) (1–3). We therefore must understand phage diversity and evolution. Here we contribute to this endeavor with the annotated genome of Vulpecula, an AS1 subcluster bacteriophage.

Vulpecula was isolated in 2022 from enriched, Vickery House Garden soil at UNG in Dahlonega, Georgia (34.53N, 83.98W) following the SEA-PHAGES protocol (4, 5). The soil was mixed with LB and incubated for 1 hour at 30°C. The supernatant was then collected and sterilized by 0.22 µm filtration. Phage presence was confirmed and purified by standard plaque assay using *A. globiformis* B-2979 and then amplified to high titer *via* web plate flooding. A Wizard DNA extraction kit (Promega) was used to produce purified genomic DNA from the lysate. A sequencing library was constructed with an NEBNext Ultra II FS kit (vers3). WGS sequencing (Illumina MiSeq) produced ~2,101× coverage from >630 k 150-base single-end reads. Overlapping terminal reads confirmed genome completeness. Newbler 2.9 (Roche) assembled the genome, and accuracy and completeness were evaluated with Consed 29 (6). The genome was found to have 37,766 bp, 67.7% GC content, and a 3′-overhang of GAGTTGCCGGGA.

Genome annotation depended on phagesdb (7) and software including DNA Master 5.23.6 (8), Glimmer 3.02 (9), GeneMark 3.26 (10), BLAST (11, 12), HHPred 2.08 (13) executed by the MPI Bioinformatics Toolkit (14), Phamerator 505 (Actino_draft) (15), tRNAscan SE 2.0 (16), Aragorn (17), and DeepTMHMM 1.0.24 (18) (default settings for all). ORFs, gaps, and potential ribosomal binding sites were first assigned with DNA Master, Glimmer, and GeneMark. Initial assignments were refined through homology assessment using Phamerator, BLAST, and HHPred. An expect (e) value <10^−4^ was used for function assignments. DeepTMHMM assessed ORFs for trans-membrane domains.

The Vulpecula genome is predicted to contain 64 open reading frames [37 with ascribed function (58%)] and no predicted tRNA genes. Genes 1–24 and 35–64 are forward oriented, and genes 25–34 are reverse oriented. Among the annotated genes are three nucleases (ORFs 29,53,64), endolysin (ORF 23), an immunity repressor (ORF 34) adjacent to tyrosine integrase (ORF 33), and an excise protein (ORF 36) as well as RusA-like resolvase (ORF 41). ORFs 14 and 15 encode overlapping tail assembly chaperones (117 and 241 aa, respectively) with ORF 14 terminated by a −1 frameshift (position 10412). ORF 28 may code for a HIP116 Rad5p N-terminal (HIRAN) domain-containing protein, which appears in numerous *Arthrobacter* species, but appears absent from other AS1 bacteriophages. Three ORFs with assigned function (ORFs 416,24) and five with unassigned function (ORFs 1,21,22,39,47) likely have transmembrane domains.

**Fig 1:**
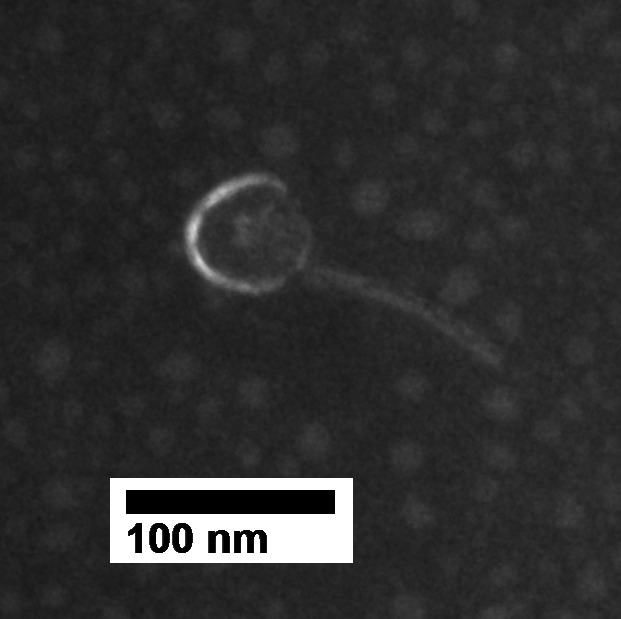
TEM image of phage Vulpecula (head diameter ~57 nm and tail length ~106 nm). The image was captured at the University of Georgia Electron Microscope facility with a JEM-1011 TEM (JOEL, Tokyo, Japan). Phosphotungstic acid was used to stain the lysate.

Genome BLAST revealed Vulpecula shares the highest nucleotide sequence similarity with *Arthrobacter* phages Ruchi (98.0% identity; OR434022.1) and Jamun (97.4% identity; OP297550.1), which were isolated in 2022 from Lumpkin County, GA (6.6 km from the Vulpecula locality) and in 2021 from Bedford, NH, respectively. Collectively, Vulpecula exhibits siphovirid morphology based on viral particle anatomy (Fig. 1) and homology assessment. Plaque morphology (7 mm circular plaques with hazy peripheries and 2 mm clear centers) and the presence of tyrosine integrase (ORF 33) support that it is temperate.

## Data Availability

The Vulpecula genome annotation can be accessed through NCBI (GenBank OR475258) and sequencing reads can be obtained from the SRA (SRX22366557).
